# Supplementary material for: Direct observation of trap-assisted recombination in organic photovoltaic devices
Source: Nat Commun. 2021 Jun 14;12:3603. doi: 10.1038/s41467-021-23870-x (PMC8203604; doi:10.1038/s41467-021-23870-x)
Supplement: Supplementary file 1 — Supplementary Information [file 41467_2021_23870_MOESM1_ESM.pdf]

Supplementary Information

Direct observation of trap-assisted recombination in organic photovoltaic devices

Stefan Zeiske[1], Oskar J. Sandberg[1,*], Nasim Zarrabi[1], Wei Li[1], Paul Meredith[1], and Ardalan Armin[1,*]

[1]Sustainable Advanced Materials (Sêr-SAM), Department of Physics, Swansea University, Singleton Park, Swansea SA2 8PP Wales, United Kingdom

*Email: o.j.sandberg@swansea.ac.uk ; ardalan.armin@swansea.ac.uk

Content:

- Supplementary Methods
  - Chemical Definitions
  - Device fabrication
  - Drift-Diffusion simulations
- Supplementary Notes
  - Supplementary Note 1: Determination of the trap depth and trap density from IPC
  - Supplementary Note 2: Trap depth estimation from combined IPC and intensity dependent V_{oc} measurements
  - Supplementary Note 3: Sub-gap absorption feature in ultra-sensitive EQE
  - Supplementary Note 4: Active layer treatment on PTB7-Th:$PC_{70}BM$
- Supplementary Figures
- Supplementary Tables
- Supplementary References

Supplementary Methods:

Chemical Definitions

PCDTBT: Poly[*N*-9′-heptadecanyl-2,7-carbazole-*alt*-5,5-(4′,7′-di-2-thienyl-2′,1′,3′-benzothiadiazole)]
$PC_{70}BM$: [6,6]-phenyl-C71-butyric acid methyl ester
m-MTDATA: 4,4',4"-Tris[(3-methylphenyl)phenylamino]triphenylamine
PTB7-Th: Poly[4,8-bis(5-(2-ethylhexyl)thiophen-2-yl)benzo[1,2-b;4,5-*b*']dithiophene-2,6-diyl-alt-(4-(2-ethylhexyl)-3-fluorothieno[3,4-b]thiophene-)-2-carboxylate-2-6-diyl)]
PBDB-T: Poly[(2,6-(4,8-bis(5-(2-ethylhexyl)thiophen-2-yl)-benzo[1,2-*b*:4,5-*b*′]dithiophene))-*alt*-(5,5-(1',3'-di-2-thienyl-5′,7′-bis(2-ethylhexyl)benzo[1′,2′-*c*:4′,5′-*c*′]dithiophene-4,8-dione)]
ITIC: 3,9-bis(2-methylene-(3-(1,1-dicyanomethylene)-indanone))-5,5,11,11-tetrakis(4-hexylphenyl)-dithieno[2,3-*d*:2′,3′-*d*′]-s-indaceno[1,2-*b*:5,6-*b*′]dithiophene
IT-4F: 3,9-bis(2-methylene-((3-(1,1-dicyanomethylene)-6,7-difluoro)-indanone))-5,5,11,11-tetrakis(4-hexylphenyl)-dithieno[2,3-*d*:2′,3′-*d*′]-s-indaceno[1,2-*b*:5,6-*b*′]dithiophene
EH-IDTBR: has not been named. CAS number: 2102510-60-9
PM6: Poly[(2,6-(4,8-bis(5-(2-ethylhexyl-3-fluoro)thiophen-2-yl)-benzo[1,2-*b*:4,5-*b*′]dithiophene))-*alt*-(5,5-(1′,3′-di-2-thienyl-5′,7′-bis(2-ethylhexyl)benzo[1′,2′-*c*:4′,5′-*c*′]dithiophene-4,8-dione)]
Y6: 2,2′-((2*Z*,2′*Z*)-((12,13-bis(2-ethylhexyl)-3,9-diundecyl-12,13-dihydro-[1,2,5]thiadiazolo[3,4-*e*]thieno[2″,3″:4′,5′]thieno[2′,3′:4,5]pyrrolo[3,2-*g*]thieno[2′,3′:4,5]thieno[3,2-*b*]indole-2,10-diyl)bis(methanylylidene))bis(5,6-difluoro-3-oxo-2,3-dihydro-1*H*-indene-2,1-diylidene))dimalononitrile
BTP-eC9: 2,2'-[[12,13-Bis(2-butyloctyl)-12,13-dihydro-3,9-dinonylbisthieno[2",3":4',5']thieno[2',3':4,5]pyrrolo[3,2-e:2',3'-g][2,1,3]benzothiadiazole-2,10-diyl]bis[methylidyne(5,6-chloro-3-oxo-1H-indene-2,1(3H)-diylidene)]]bis[propanedinitrile]
PEDOT:PSS: Poly(3,4-ethylenedioxythiophene) polystyrene sulfonate
PDINO: 2,9-bis[3-(dimethyloxidoamino)propyl]anthra[2,1,9-def:6,5,10-*d*′*e*′*f*′]diisoquinoline-1,3,8,10(2*H*,9*H*)-tetrone

Device fabrication

PCDTBT:$PC_{70}BM$, PCDTBT:$PC_{70}BM$:m-MTDATA, PTB7-Th:$PC_{70}BM$ (w/ & w/o DIO), PTB7-Th:ITIC, PBDB-T:ITIC, PBDB-T:IT-4F, PBDB-T:EH-IDTBR, and PM6:IT-4F solar cells were fabricated with an inverted architecture (Glass/ Indium Tin Oxide (ITO)/ZnO/Active Layer/MoO_3/Ag). The commercial ITO patterned glass electrodes were cleaned by sonication in distilled water, acetone and 2-propanole in sequence each for 10 minutes. The cleaned substrates were first dried with a stream of nitrogen and then transferred to a 100 °C hotplate, and further treated with an Oxygen plasma for 10 min. The ZnO performs as an electron transport layer and was prepared by dissolving 200 mg zinc acetate dihydrate (purchased from Sigma Aldrich) in 2-methoxyethanol (2 mL) using ethanolamine (56 µL) as the stabilizer. The

solution was stirred overnight under ambient conditions and spin-coated (4000 rpm for 30s) onto the ITO substrates and further annealed at 200 °C for 1 hour to obtain a thickness of approximately 30 nm. Active layer deposition of the above systems was conducted by spin-casting, and their details are listed below. Subsequently, 7 nm of MoO_3 and 100 nm of Ag were evaporated through a shadow mask in a vacuum chamber with $<10^{-6}$ mbar base pressure defining a 0.04 cm^2 cell area for each pixel. Afterwards, devices were sealed with a cover glass using UV light-annealed glue from Bluefix. All the thicknesses of the above films are measured by ellipsometry.

Details of active layer depositions are as follows:

PCDTBT:$PC_{70}BM$: PCDTBT and $PC_{70}BM$ were purchased from Ossila. A total concentration of 25 mg mL^{-1} in Chlorobenzene (CB) with a donor: acceptor ratio of 1:4 was used to prepare the active layer solution. The solution was spin-coated at 1500 rpm to form a film with the thickness around 100 nm.

PCDTBT:$PC_{70}BM$:m-MTDATA (1 %): A total concentration of 25 mg mL^{-1} in CB with a PCDTBT:$PC_{70}BM$:m-MTDATA weight ratio of 1:4:0.01 was used to prepare the active layer solution. The solution was spin-coated at 1500 rpm to form a film with the thickness around 100 nm.

PTB7-Th:$PC_{70}BM$ (w/ DIO): PTB7-Th was purchased from Zhi-yan (Nanjing). A total concentration of 16 mg/ml in CB:DIO (97:3, v/v) with a donor: acceptor ratio of 1:1.5 was used to prepare the active layer solution. The solution was spin-coated at 600 rpm to form a film with the thickness around 110 nm.

PTB7-Th:$PC_{70}BM$ (w/o DIO): A total concentration of 16 mg/ml in CB solution with a donor: acceptor ratio of 1:1.5 was used to prepare the active layer solution. The solution was spin-coated at 600 rpm to form a film with the thickness around 110 nm.

PTB7-Th:ITIC: ITIC was purchased from Zhi-yan (Nanjing). A total concentration of 14 mg/ml in CB:DIO (99:1, v/v) with a donor: acceptor ratio of 1:1.4 was used to prepare the active layer solution. The solution was spin-coated at 700 rpm to form a film with the thickness around 100 nm.

PBDB-T:ITIC: PBDB-T was purchased from Zhi-yan (Nanjing). A total concentration of 16 mg/ml in CB:DIO (99:1, v/v) with a donor: acceptor ratio of 1:1 was used to prepare the active layer solution. The solution was spin-coated at 900 rpm and further thermal annealed at 100 °C for 10 min to form a film with the thickness around 100 nm.

PBDB-T:IT-4F: IT-4F was purchased from Solarmer (Beijing). A total concentration of 16 mg/ml in CB:DIO (99:1, v/v) with a donor: acceptor ratio of 1:1 was used to prepare the active layer solution. The solution was spin-coated at 900 rpm and further thermal annealed at 100 °C for 10 min to form a film with the thickness around 100 nm.

PBDB-T:EH-IDTBR: EH-IDTBR was purchased from Solarmer (Beijing). A total concentration of 16 mg/ml in CB:DIO (99:1, v/v) with a donor: acceptor ratio of 1:1 was used to prepare the active layer solution. The solution was spin-coated at 900 rpm and further thermal annealed at 100 °C for 10 min to form a film with the thickness around 100 nm.

PM6:IT-4F: A total concentration of 14 mg/ml in Chloroform (CF): DIO (99.5:0.5, v/v) with a donor: acceptor ratio of 1:1 was used to prepare the active layer solution. The solution was spin-coated at 3000 rpm and further thermal annealed at 100 °C for 10 min to form a film with thickness around 100 nm.

PM6:ITIC, PBDB-T:Y6, PM6:Y6 and PM6:BTP-eC9 solar cells were fabricated with an conventional architecture (Glass/ Indium Tin Oxide (ITO)/PEDOT:PSS/Active Layer/PDINO/Ag). The commercial ITO patterned glass electrodes were cleaned by sonication in distilled water, acetone and 2-propanole in sequence each for 10 minutes. The cleaned substrates were first dried with a stream of nitrogen and then transferred to a 100 ºC hotplate, and further treated with an Oxygen plasma for 10 mins. PEDOT: PSS solution was first diluted with the same volume of water and then cast at 4000 rpm on ITO substrates followed by thermal annealing at 155 ºC for 15 min to form a 10 nm film. Active layer deposition of the above systems was conducted by spin-casting, and their details are listed below. Subsequently, 1.5 mg ml^{-1} PDINO solution was spin-coated on active layer film at 2000 rpm to form 10 nm films, and 100 nm of Ag were evaporated through a shadow mask in a vacuum chamber with $<10^{-6}$ mbar base pressure. Afterwards, devices were sealed with a cover glass using UV light-annealed glue from Bluefix. All the thickness of the above films are measured by ellipsometry.

PM6:ITIC: PM6 was purchased from Solarmer (Beijing). A total concentration of 14 mg/ml in CF:DIO (99:1, v/v) with a donor: acceptor ratio of 1:1 was used to prepare the active layer solution. The solution was spin-coated at 3000 rpm and further thermal annealed at 100 ºC for 10 min to form a film with the thickness around 100 nm.

PBDB-T:Y6: Y6 was purchased from Solarmer (Beijing). A total concentration of 12 mg/ml in CF: 1-chloronaphthalene (99.5:0.5, v/v) with a donor: acceptor ratio of 1:1.2 was used to prepare the active layer solution. The solution was spin-coated at 2000 rpm and further thermal annealed at 100 ºC for 10 min to form a film with the thickness around 100 nm.

PM6:Y6: A total concentration of 16 mg/ml in CF: 1-chloronaphthalene (99.5:0.5, v/v) with a donor: acceptor ratio of 1:1.2 was used to prepare the active layer solution. The solution was spin-coated at 3000 rpm and further thermal annealed at 100 ºC for 10 min to form a film with the thickness around 100 nm.

PM6:BTP-eC9: BTP-eC9 was purchased from Solarmer (Beijing). A total concentration of 16 mg/ml in CF: DIO (99.5:0.5, v/v) with a donor: acceptor ratio of 1:1.2 was used to prepare the active layer solution. The solution was spin-coated at 3000 rpm and further thermal annealed at 100 ºC for 10 min to form a film with the thickness around 100 nm.

PTB7-Th:$PC_{70}BM$ hole-only and electron-only devices: The device structures of hole-only and electron-only devices were: ITO/PEDOT:PSS/active layer/MoO_3/Ag and ITO/ZnO/active layer/PDINO/Ag respectively. For the hole-only devices, 40 nm PEDOT:PSS films were cast on clean ITO substrate. For the deposition of the PTB7-Th:$PC_{70}BM$ active layer, 1400 nm PTB7-Th:$PC_{70}BM$ (1:1.5, w/w) film was spin-coated (500 rpm) on PEDOT:PSS or ZnO substrates from CB solutions (50mg mL^{-1} with 3 vol% DIO) and further rinsed with 80 µL of methanol at 4000 rpm for 20 s. Following this, 7 nm MoO_3 and 100 nm Ag were thermally evaporated to form a cathode under high vacuum. For the electron-only device, a 30 nm ZnO film was cast on clean ITO substrates. Subsequently, the active layer was cast onto the ZnO substrate with the same method as the hole-only device. Following this, an 8 nm PDINO film was spin-coated (3000 rpm) from methanol solution (1mg mL^{-1}), and 100 nm of Ag thermally evaporated on PDINO to form an anode.

PBDB-T:ITIC hole-only and electron-only devices: The device structures of the hole-only and electron-only devices, and the deposition methods of PEDOT:PSS, ZnO, PDINO, MoO_3 and

Ag were the same as the PTB7-Th:$PC_{70}BM$ hole-only and electron-only devices. For the deposition of the PBDB-T:ITIC active layer, a 800 nm PBDB-T: ITIC (1:1, w/w) film was spin-coated (1200 rpm) on PEDOT:PSS or ZnO substrates from CB solutions (50 mg mL^{-1} with 1 vol% DIO) and further thermal annealed at 100 °C for 10 min.

PCDTBT:$PC_{70}BM$ hole-only and electron-only devices: The device structures of the hole-only and electron-only devices, and the deposition methods of PEDOT:PSS, ZnO, PDINO, MoO_3 and Ag were the same as the PTB7-Th:$PC_{70}BM$ hole-only and electron-only devices. For the deposition of the PCDTBT:$PC_{70}BM$ active layer, 1100 nm PCDTBT:$PC_{70}BM$ (1:4, w/w) film was spin-coated (500 rpm) on PEDOT:PSS or ZnO substrates from CB solutions (50 mg mL^{-1}).

Drift-Diffusion simulations

A numerical drift-diffusion model was used for the device simulations. The model solves the electron and hole current density equations, coupled to the Poisson equation and the steady-state continuity equations for electrons and holes, respectively:

$$J_\mathrm{n} = -qn\mu_\mathrm{n}\frac{d\psi}{dx} + \mu_\mathrm{n}kT\frac{dn}{dx} \quad (1)$$

$$J_\mathrm{p} = -qp\mu_\mathrm{p}\frac{d\psi}{dx} - \mu_\mathrm{p}kT\frac{dp}{dx} \quad (2)$$

$$\frac{d^2\psi}{dx^2} = -\frac{q}{\varepsilon\varepsilon_0}[p - n - n_\mathrm{t}] \quad (3)$$

$$-\frac{1}{q}\frac{dJ_n}{dx} = G(x) - \beta[np - n_\mathrm{i}^2] - R_\mathrm{SRH} \quad (4)$$

$$\frac{1}{q}\frac{dJ_\mathrm{p}}{dx} = G(x) - \beta[np - n_\mathrm{i}^2] - R_\mathrm{SRH} \quad (5)$$

where x is the distance from the anode contact into the active layer. Here, ψ is the electrical potential, n is the electron density and p is the hole density, while μ_n (μ_p) is the electron (hole) mobility, G is the optical generation rate of free electrons and holes, and β is the bimolecular ("band-to-band") recombination coefficient, with $n_\mathrm{i}^2 = N_\mathrm{c}N_\mathrm{v}\exp\left(-\frac{E_\mathrm{g}}{kT}\right)$; N_v and N_c is the effective density of transport states in the donor and acceptor, respectively, while E_g is the associated D-A bandgap. Furthermore, q is the elementary charge, k the Boltzmann constant, T the absolute temperature, ε_0 the vacuum permittivity and ε is the relative permittivity of the active layer. Finally, n_t is the density of trapped electrons and R_SRH is the trap-assisted recombination rate.

The traps are assumed to be acceptor-like (or electron traps), thus contributing to the total negative space charge when occupied by electrons. We note, however, that owing to the assumed symmetry between electrons and holes in the simulations (in terms of mobilities and the contacts), the case with donor-like (hole) traps (traps are neutral when occupied by an electron and positively charged when occupied by a hole) will yield completely identical results. The occupation of traps and trap-assisted recombination is described in accordance with Shockley-Read-Hall (SRH) statistics,[1]

$$n_\mathrm{t} = \frac{N_t[\tau_\mathrm{p}n + \tau_\mathrm{n}p_1]}{\tau_\mathrm{p}[n+n_1] + \tau_\mathrm{n}[p+p_1]} \quad (6)$$

$$R_{\mathrm{SRH}} = \frac{[np - n_{\mathrm{i}}^2]}{\tau_{\mathrm{p}}[n + n_1] + \tau_{\mathrm{n}}[p + p_1]} \tag{7}$$

where N_{t} is the trap density, τ_{n} (τ_{p}) is the associated SRH lifetime for electrons (holes), while

$$n_1 = N_{\mathrm{c}} \exp\left(-\frac{\Delta_{\mathrm{t}}}{kT}\right) \tag{8}$$

$$p_1 = N_{\mathrm{v}} \exp\left(-\frac{E_{\mathrm{g}} - \Delta_{\mathrm{t}}}{kT}\right) \tag{9}$$

with Δ_{t} being the trap depth.

These equations are solved numerically for $\psi(x)$, $n(x)$ and $p(x)$ using the Scharfetter-Gummel discretization method and Gummel's iteration scheme; see Ref. [1]. This numerical approach has been shown to be very reliable and robust. We assume an active layer of thickness d, where $x = 0$ and $x = d$ corresponds to the anode and cathode contact, respectively. Thermal equilibrium is assumed to apply for charge carriers at the contacts (large surface recombination velocities); the corresponding six boundary conditions are given by

$$\psi(0) = V_{\mathrm{dev}} - U_{\mathrm{BI}} \tag{10}$$

$$\psi(d) = 0 \tag{11}$$

$$p(0) = N_{\mathrm{v}} \exp\left(-\frac{\varphi_{\mathrm{an}}}{kT}\right) \tag{12}$$

$$n(d) = N_{\mathrm{c}} \exp\left(-\frac{\varphi_{\mathrm{cat}}}{kT}\right) \tag{13}$$

$$p(d) = \frac{n_{\mathrm{i}}^2}{n(d)} \tag{14}$$

$$n(0) = \frac{n_{\mathrm{i}}^2}{p(0)} \tag{15}$$

where $U_{\mathrm{BI}} = [E_{\mathrm{g}} - \varphi_{\mathrm{an}} - \varphi_{\mathrm{cat}}]/q$ is the built-in voltage and V_{dev} is the applied voltage across the active layer. φ_{cat} and φ_{an} is the injection barrier for electrons at the cathode and holes at the anode contact, respectively. The discretized Poisson equation, electron continuity equation, and hole continuity equation are written as three matrix equations of the form $\bar{\bar{A}}_{\alpha}\bar{y}_{\alpha} = \bar{b}_{\alpha}$ for $\alpha = \psi, n, p$, where $\bar{y}_{\alpha}^T = [\alpha(x_1), \alpha(x_2), \dots]$. The subsequent tridiagonal matrix systems are solved using the C-routine *tridag* from Ref [2], finally allowing for the device current $J_{\mathrm{dev}} = J_{\mathrm{n}} + J_{\mathrm{p}}$ to be evaluated.

The total current density, which is composed of the device current J_{dev} flowing in parallel with the leakage current induced by a finite shunt resistance R_{sh}, is then obtained from

$$J(V) = J_{\mathrm{dev}}(V_{\mathrm{dev}}) + \frac{V_{\mathrm{dev}}}{R_{\mathrm{sh}}} \tag{16}$$

$$V_{\mathrm{dev}} = V - JR_{\mathrm{s}} \tag{17}$$

where V is the external applied voltage and R_{s} is the external series resistance. In the simulations, we assume equal effective density of states for free electrons and holes and equal injection barriers at both contacts. Furthermore, balanced electron and hole mobilities are assumed. The SRH lifetimes for electrons and holes are taken to be equal: $\tau_{\mathrm{n}} = \tau_{\mathrm{p}} = \tau_{\mathrm{SRH}}$. The simulation parameters have been listed in Supplementary Table 2. Finally, an optical transfer-

matrix model, which accounts for wavelength-dependent interference effects and multiple reflections in the device stack, is used to obtain realistic photogeneration rate profiles for free charge carriers, using the complex refractive indices of the different layers in the device stack as input.[3,4]

Supplementary Notes:

Supplementary Note 1: Determination of the trap depth and trap density from IPC

The trap depth can be estimated from the point-of-transition (POT) between the low intensity regime, where first-order SRH recombination in the bulk is negligible, and the moderate intensity regime where first-order SRH recombination dominates (trap-filling limit). We consider a device with an active layer containing electron traps (acceptor-type); the case with hole traps is analogous. At low intensities (when recombination and space charge effects are negligible), the associated average electron density n_{low} is related to the total short-circuit current J_{sc} via

$$J_{\mathrm{sc}} = 2qn_{\mathrm{low}}\mu_{\mathrm{n}}F \equiv 2qn_{\mathrm{low}}d/t_{\mathrm{tr}} \quad (18)$$

where t_{tr} is the electron transit time (μ_{n} is the electron mobility and F the electric field). At these intensities, we also expect $J_{\mathrm{sc}} \approx J_{\mathrm{G}}$, where J_{G} is the photogeneration current. POT occurs when $n_{\mathrm{low}} \approx n_1$ (see main text); making use of the definition of n_1, it then follows that

$$\Delta_{\mathrm{t}} = kT \ln\left(2qN_{\mathrm{L,A}}d/\left[J_{\mathrm{G,POT}}t_{\mathrm{tr}}\right]\right) \quad (19)$$

allowing for the trap depth Δ_{t} to be determined. This analytical expression is verified by numerical drift-diffusion simulations, as demonstrated in Supplementary Fig. 8.

To obtain an order of magnitude estimate of the trap density N_{t}, in turn, we consider the moderate intensity regime. In this regime, the occupied traps will behave as fixed negative charges. In analogy to the case with p-type doping (where the fixed negative charges are ionized dopants)[5], the electric field inside the active layer will be mainly concentrated to a space charge region of trapped electrons (adjacent to the cathode), while the remaining active layer is neutral (the trapped electrons are neutralized by injected/generated free holes) with $F \approx 0$. Then, assuming that the charge extraction of photo-generated carriers is predominantly taking place from the space charge region, we expect the charge collection efficiency under short-circuit conditions to take the approximate form[5]:

$$\eta_{\mathrm{col}} \sim \frac{w_{\mathrm{sc}}}{d} \quad (20)$$

where

$$w_{\mathrm{sc}} = \sqrt{\frac{2\varepsilon\varepsilon_0 U_{\mathrm{bi}}}{qN_{\mathrm{sc}}}} \quad (21)$$

is the space charge region thickness and d is the active layer thickness. Here, N_{sc} is the space charge density (of trapped electrons) within the space charge region, while U_{bi} is the associated built-in potential. Assuming $N_{\mathrm{sc}} \approx N_{\mathrm{t}}$, and noting that $\eta_{\mathrm{col}} \approx \mathrm{EQE}_{\mathrm{norm}}$, the following expression for estimating the trap density is obtained:

$$N_{\mathrm{t}} \sim \frac{2\varepsilon\varepsilon_0 U_{\mathrm{bi}}}{qd^2} \times \frac{1}{\mathrm{EQE}_{\mathrm{norm}}^2} \quad (22)$$

where $\mathrm{EQE}_{\mathrm{norm}}$ is the EQE of the second EQE plateau at moderate irradiance normalized to the EQE plateau at low irradiance (with higher magnitude).

Supplementary Note 2: Trap depth estimation from combined IPC and intensity dependent V_{oc} measurements

The energy of the trap states, located within the donor-acceptor bandgap E_g (with $E_g = |E_{H,D} - E_{L,A}|$, where $E_{H,D}$ denotes the HOMO energy level of the organic donor and $E_{L,A}$ denotes the LUMO energy level of the organic acceptor), can be determined if the point-of-transition is known. Assuming a symmetric quasi-Fermi level splitting E_{QFLS} at the point of transition (i.e., $E_{QFLS} = |E_{F,n} - E_{F,p}|$ around the mid-gap of the organic D-A BHJ, the trap depth (relative to the HOMO energy level of the donor, respectively LUMO energy level of the acceptor) can be estimated from

$$\Delta_{t,QFLS} = \frac{E_g - E_{QFLS}}{2} = \frac{|E_{H,D} - E_{L,A}| - E_{QFLS}}{2}. \quad (23)$$

Provided that surface recombination is negligible, the V_{oc} of an organic solar cell gives a direct estimate of the quasi-Fermi level splitting $E_{QFLS} = qV_{oc}$ in the bulk. However, at low light intensities, the V_{oc} (and subsequently the determination of E_{QFLS}) becomes limited by the shunt resistance of the device. In such cases an extrapolation of the V_{oc} from high to low intensity may be used to estimate a would-be-shunt-free V_{oc} at lower light intensities in order to obtain E_{QFLS}. This is demonstrated in Supplementary Fig. 1a for PCDTBT:PC70BM, where $E_{QFLS} = 0.57$ eV (horizontal, dotted line) is obtained at the point-of-transition (POT; vertical dotted line). With a donor HOMO energy level (PCDTBT) of $E_{H,D} = -5.3$ eV and an acceptor LUMO energy level (PC70BM) of $E_{L,A} = -3.91$ eV (values are taken from literature, see Supplementary Table 1) it follows that $\Delta_{t,QFLS} \approx 0.41$ eV. This value is close to the obtained $\Delta_{t,IPC} \approx 0.48$ eV based on IPC. Supplementary Fig. 1b shows the consistency between the determined trap depths via IPC ($\Delta_{t,IPC}$) and open-circuit voltage ($\Delta_{t,QFLS}$) approach for all OPVs studied in this work. The solid (dashed) line indicates $\Delta_{t,QFLS} = \Delta_{t,IPC}$ (± 0.1 eV).

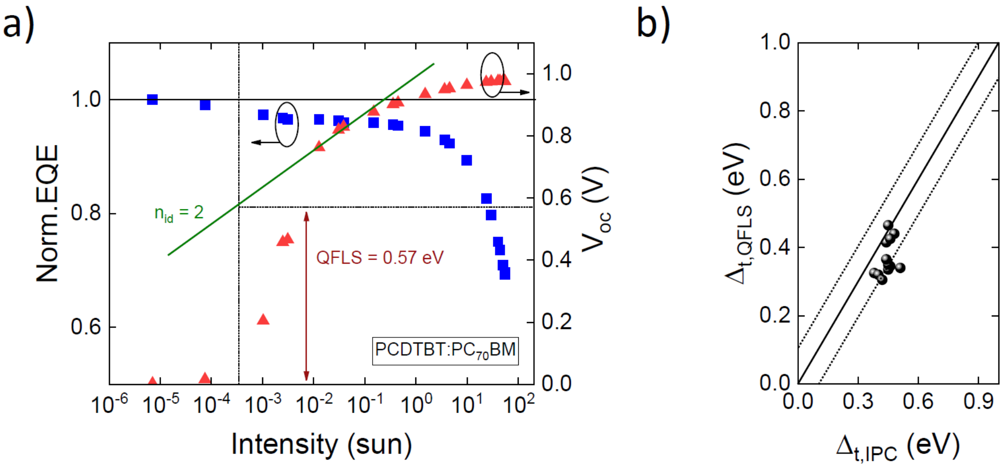

Figure 1 a) Normalized external quantum efficiency (EQE) (left axis) and open-circuit voltage (V_{oc}) (right axis) of a PCDTBT:PC70BM solar cell plotted as a function of irradiance. The vertical dotted line marks the intensity at the point-of-transition between the two EQE plateaus, where $E_{F,n} = E_t$. The corresponding quasi-Fermi level splitting was estimated to 0.57 eV from the would-be shunt-free V_{oc} corresponding to the ideality factor of $n_{id} = 2$ (green line). A trap depth of $\Delta_{t,QFLS} = 0.41$ eV was calculated. **b)** Comparison between trap depth estimated from IPC (i.e., via $\Delta_{t,IPC} = kT \ln\left(2qN_{L,A}d/[J_{G,POT}t_{tr}]\right)$ as described in the main text) and via quasi-Fermi level splitting at the point-of-transition obtained from the open-circuit voltage. For comparison, $\Delta_{t,QFLS} = \Delta_{t,IPC}$ ($\pm$ 0.1 eV) is indicated by the black solid line (dashed lines).

Supplementary Note 3: Sub-gap absorption feature in ultra-sensitive EQE

For the analysis of the ultra-sensitive EQE spectra, in line with Marcus charge-transfer, double-Gaussian functions (red solid lines) is used to fit the reduced EQE (i.e. EQE times photon energy; EQE_{red}) at photon energies well below the gap accounting for both CTS and trap state absorption features. The associated fit function is given by[6]

$$\mathrm{EQE}_{\mathrm{red}}(h\nu) = \frac{A_{\mathrm{CTS}}}{\sigma_{\mathrm{CTS}}\sqrt{2\pi}} \exp\left(-\frac{(h\nu - E_{\mathrm{CTS}})^2}{2\sigma_{\mathrm{CTS}}^2}\right) + \frac{A_{\mathrm{trap}}}{\sigma_{\mathrm{trap}}\sqrt{2\pi}} \exp\left(-\frac{(h\nu - E_{\mathrm{trap}})^2}{2\sigma_{\mathrm{trap}}^2}\right) \quad (24)$$

where $h\nu$ is the photon energy. Here, σ is related to the width (which depends on the reorganization energy), A is a constant, while E denotes the peak position. As can be seen in Fig. 3c in the main text, while the CTS absorption feature (Gaussian shape according to Marcus model[7]) remains the same for the PCDTBT:$PC_{70}BM$ and PCDTBT:$PC_{70}BM$:m-MTDATA device, the trap feature significantly increases when adding m-MTDATA to the neat PCDTBT:$PC_{70}BM$.

Supplementary Note 4: Active layer treatment on PTB7-Th:$PC_{70}BM$

It has been reported that PTB7-Th:$PC_{70}BM$ solar cells processed with CB solution or CB:DIO solution could render contrasting morphology and defects in active layer. Subsequently, PTB7-Th:$PC_{70}BM$ (w/ DIO) and PTB7-Th:$PC_{70}BM$ (w/o DIO) devices were fabricated (details are provided in the Device fabrication section). The corresponding current density versus applied voltage characteristics, under artificial 1 sun condition, and normalized EQE, based on intensity dependent photocurrent (IPC) measurements, are shown in Supplementary Fig. 2a and Fig. 2b, respectively. It has been suggested that the different processing methods are linked to different morphology in the active layer.[8–12] Based on IPC, an increased second-order recombination in PTB7-Th:$PC_{70}BM$ processed without DIO can be discerned, consistent with the drastic reduction in the Fill Factor. In contrast, the differences in the trap depth and trap density for PTB7-Th:$PC_{70}BM$ with and without DIO were found to be negligible (see Supplementary Table 1). This points towards a trap origin that is not directly linked to morphology.

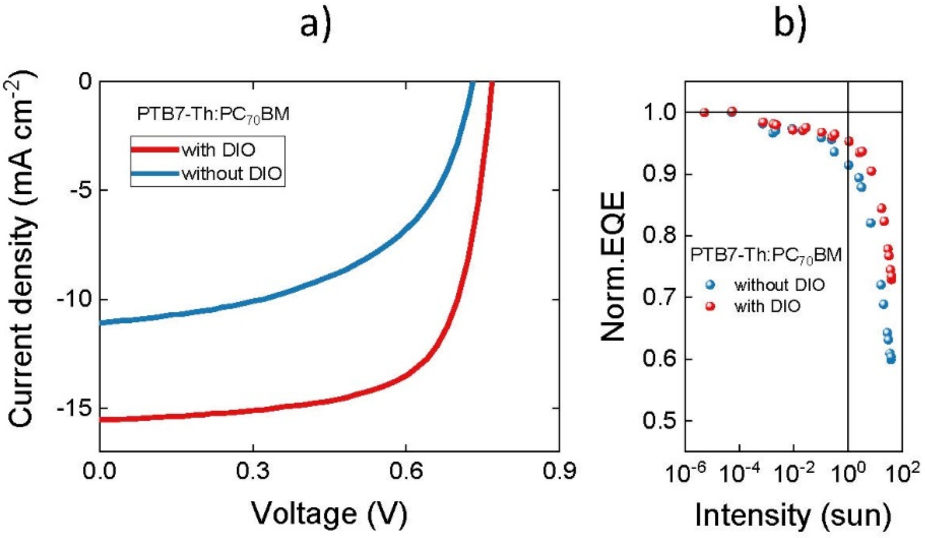

Figure 2 (a) Current-density versus applied voltage under artificial AM1.5G condition compared for a PTB7-Th:$PC_{70}BM$ device with (red) and without (blue) DIO. (b) Corresponding external quantum efficiency (EQE) of PTB7-Th:$PC_{70}BM$ with DIO plotted as a function of intensity. The excitation wavelength was set to $\lambda = 520$ nm and no bias voltage was applied on the devices. The EQE was calculated from the measured short-circuit current

density (J_{sc}) and irradiance (I) via EQE $= \frac{J_{sc}}{I} \times \frac{hc}{\lambda}$, where h is the Planck constant and c the speed of light. (c) Repetition of panel (b), but for a PTB7-Th:$PC_{70}BM$ solar cell without DIO.

Supplementary Figures

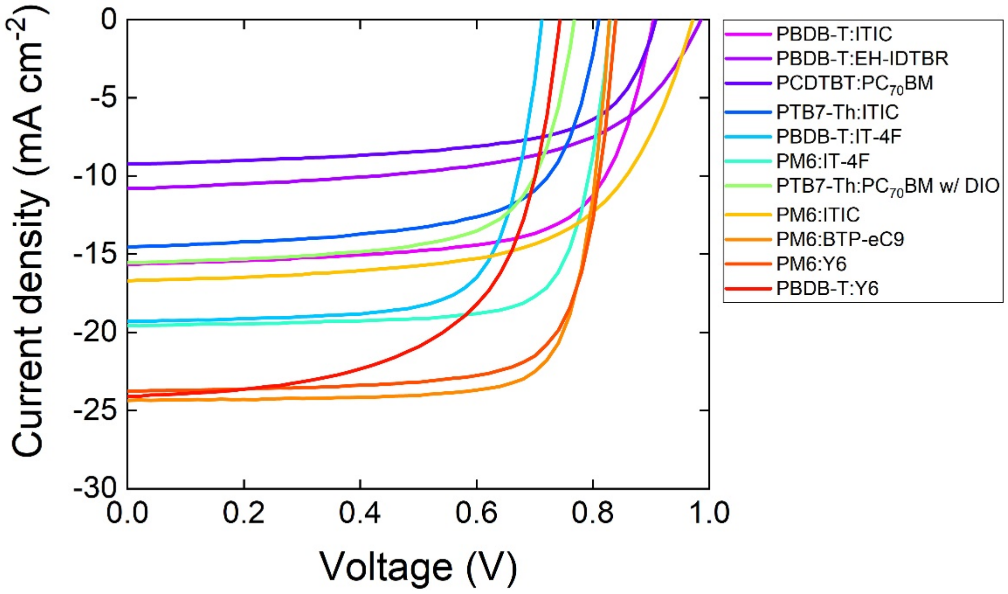

Figure 3 Current density as a function of applied bias voltage of different fullerene and non-fullerene acceptor based organic solar cells measured under artificial AM1.5G conditions. No hysteresis or dependence on the scan speed was observed.

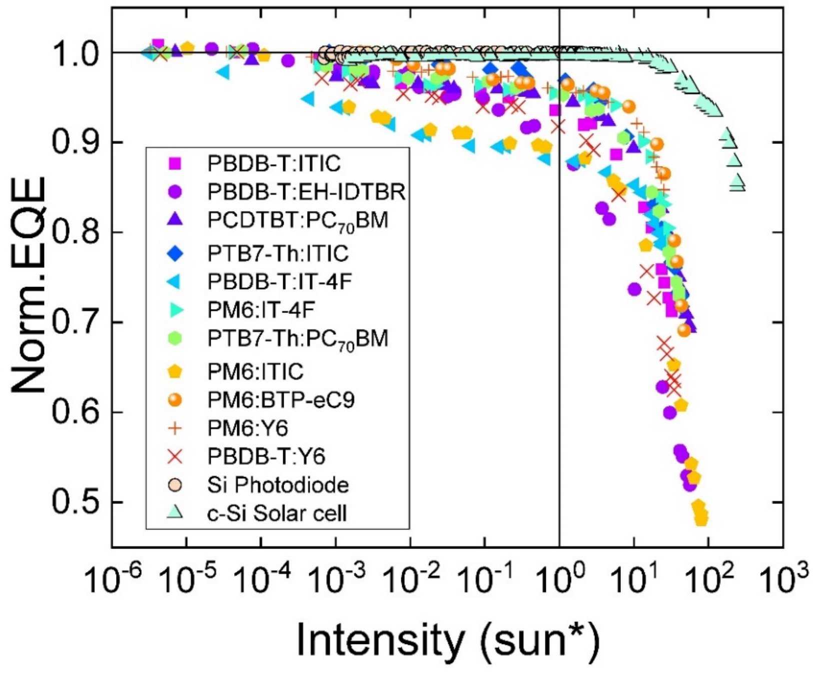

Figure 4 Normalized external quantum efficiency (EQE) of a large variety of organic solar cells plotted as a function of intensity and compared with inorganic crystalline silicon solar cell (c-Si) and photodiode sensor. The excitation wavelength was set to $\lambda = 520$ nm and no bias voltage was applied on the devices (short-circuit condition). The vertical line marks the 1 sun equivalent light intensity at 520 nm. The EQE was calculated from the measured short-circuit current density (J_{sc}) and irradiance (I) via EQE $= \frac{J_{sc}}{I} \times \frac{hc}{\lambda}$, where h is the Planck constant and c the speed of light.

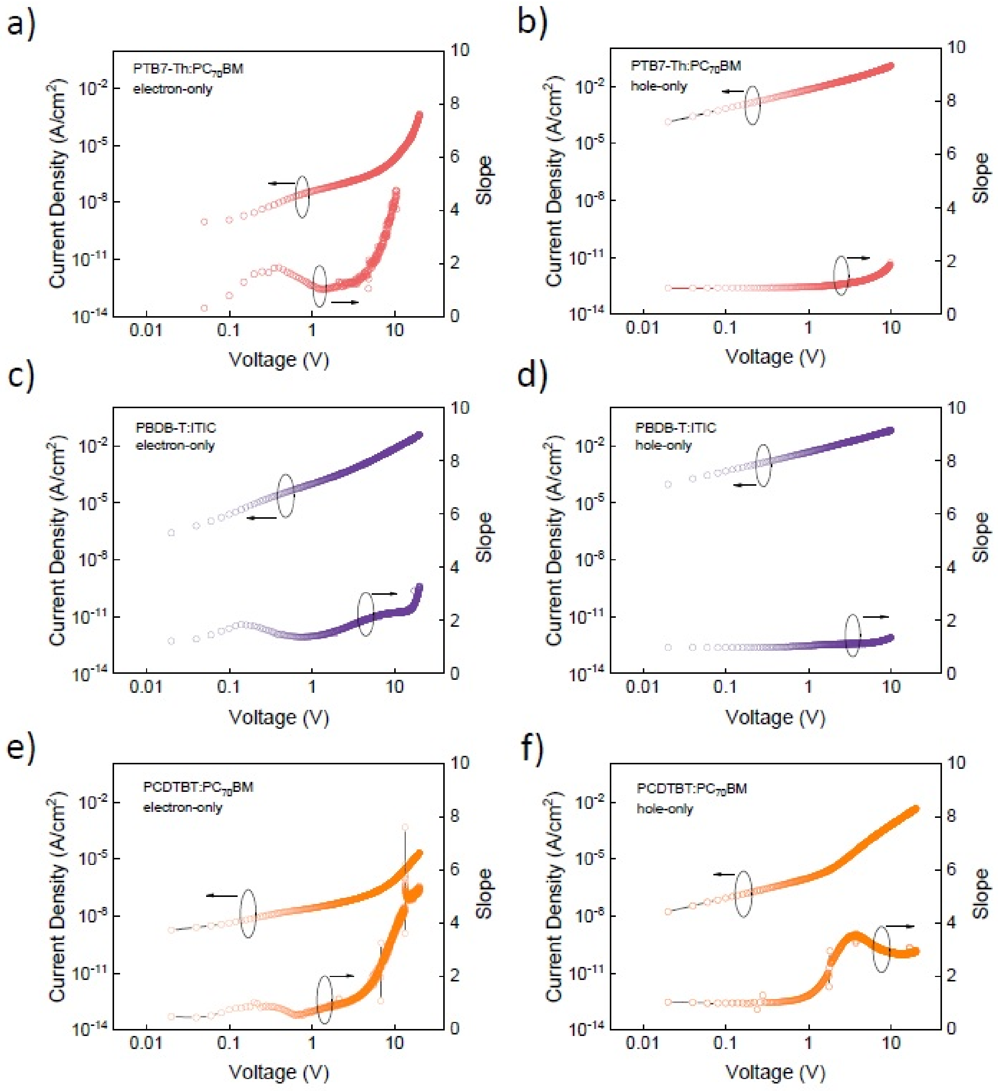

Figure 5 Current density (left axis) and calculated slope (right axis) versus applied bias voltage of a 1400 nm thick PTB7-Th:PC_{70}BM, 800 nm thick PBDB-T:ITIC and 1100 nm thick PCDTBT:PC_{70}BM electron-only (left column) and hole-only (right column) device. The weak hump in the slope of the PTB7-Th:PC_{70}BM and PBDB-T:ITIC electron-only (PCDTBT:PC_{70}BM hole-only) device reveal electron (hole) trap states in the donor: acceptor bulk.[13,14]

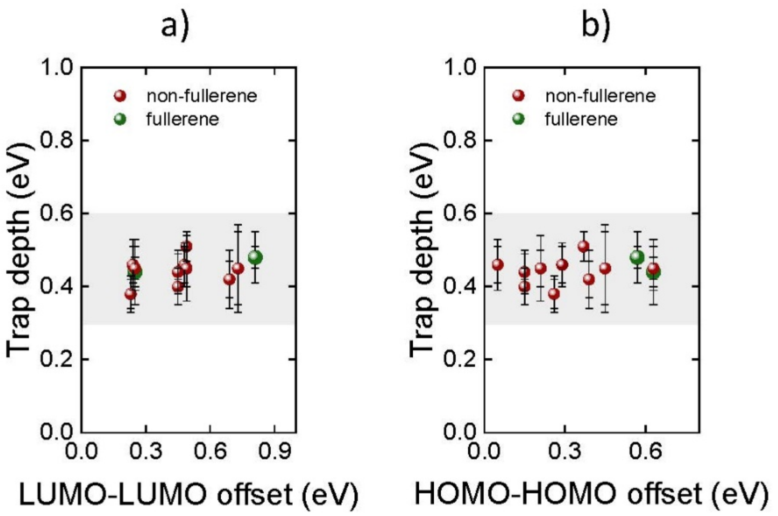

Figure 6 Trap depth of fullerene (green) and non-fullerene (red) acceptor based organic solar cells plotted against the (a) LUMO-LUMO offset and (b) HOMO-HOMO offset. The trap depths (and error bars) were determined via IPC as described in the main text. The values of HOMO and LUMO energy levels are taken from literature and are provided in Supplementary Table 1.

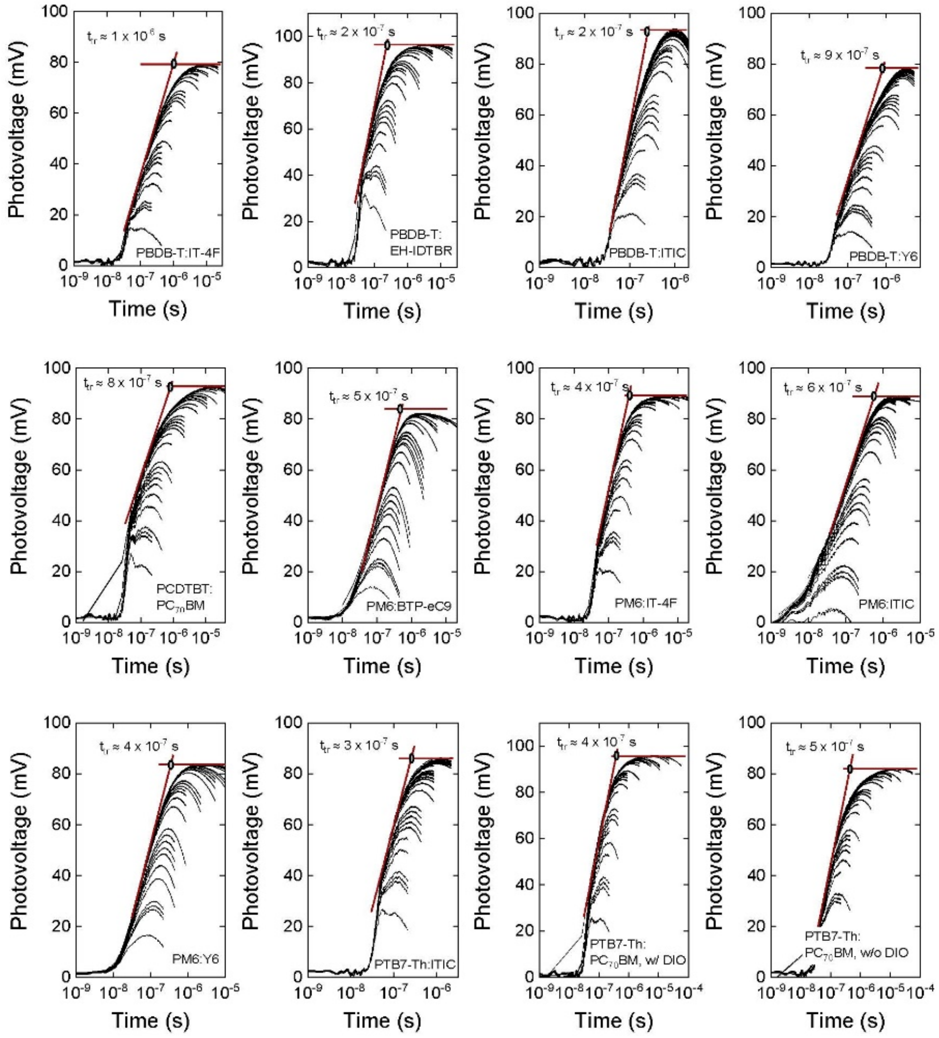

Figure 7 Resistance-dependent photovoltage (RPV) signals of a large variety of organic solar cell. The load resistance was stepwise varied between 50 Ω and 1 MΩ changing the magnitude of the transient signal.

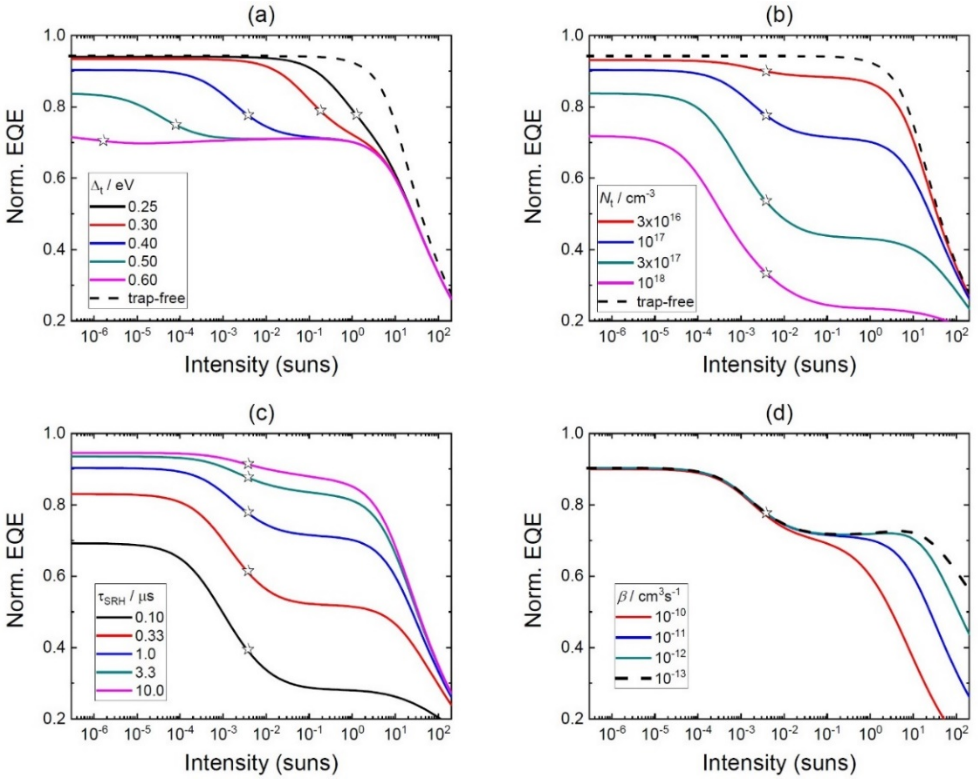

Figure 8 Normalized and simulated external quantum efficiency (EQE) plotted as a function of light intensity at varying (a) trap depth (Δ_{t}), (b) trap density (N_{t}), (c) Shockley-Read-Hall carrier lifetime (τ_{SRH}), and (d) bimolecular recombination coefficient (β). Star-shaped symbols mark the analytically expected POTs (see Supplementary Note 1) between the two EQE plateaus at low and moderate light intensity. Model parameters are, unless explicitly varied, the same as in Figure 1 in the main manuscript; see Supplementary Table 2.

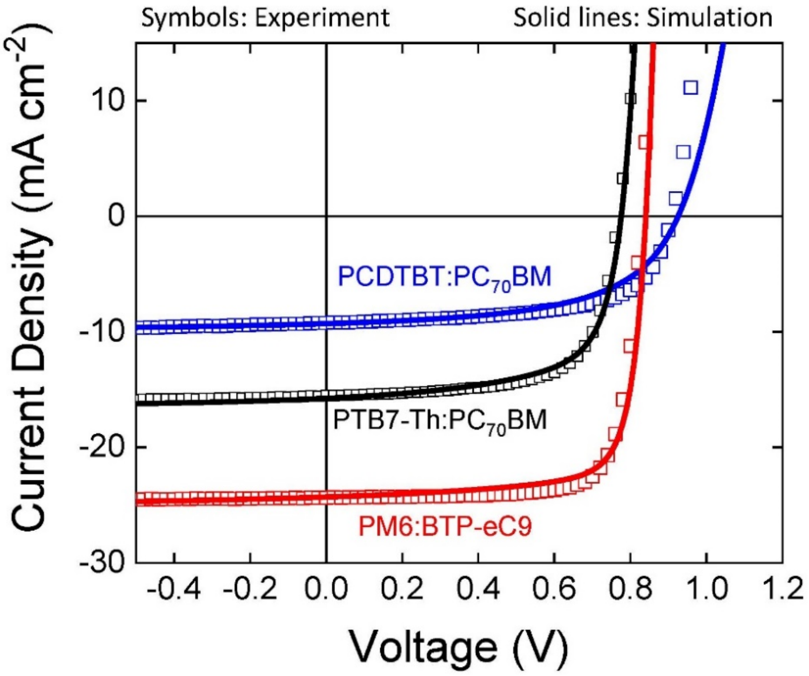

Figure 9 Experimentally obtained (symbols) and simulated (solid lines) current density versus applied voltage curves of PCDTBT:PC_{70}BM (blue), PTB7-Th:PC_{70}BM (black) and PM6:BTP-eC9 (red) under artificial AM 1.5G conditions. Model parameters are provided in Supplementary Table 2. No hysteresis or dependence on the scan speed was observed.

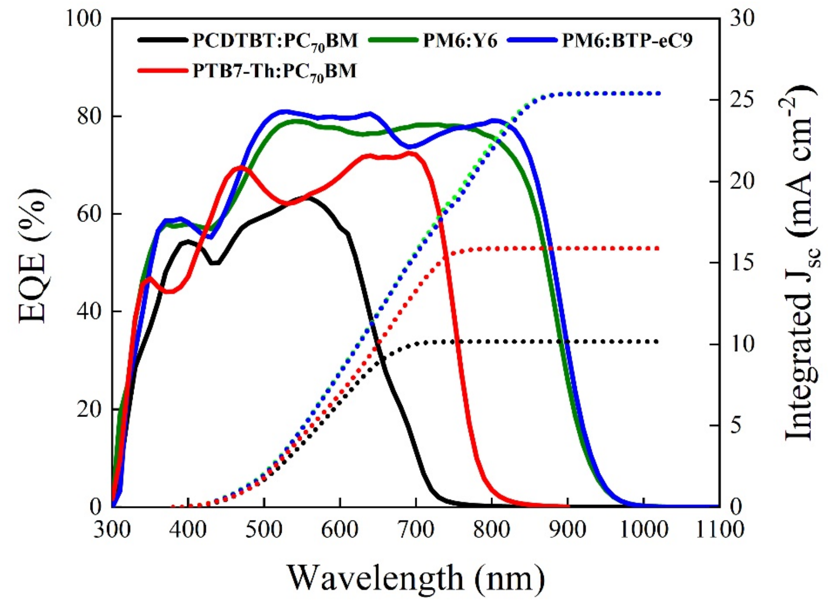

Figure 10 External quantum efficiency (EQE) spectra (left axis; solid lines) and integrated short-circuit current density, J_{sc}, (right axis; dotted lines) plotted as a function of wavelength for PCDTBT:PC_{70}BM, PM6:Y6, PTB7-Th:PC_{70}BM, and PM6:BTP-eC9 solar cell devices. The integrated J_{sc} are consistent with the J_{sc} measured under AM 1.5G conditions within 5% error. No spectral mismatch correction was therefore required in the J-V measurements.

Supplementary Tables

Table 1 Energy of donor HOMO ($E_{\mathrm{H,D}}$) and acceptor LUMO ($E_{\mathrm{L,A}}$) levels, active layer thicknesses (d), trap depth ($\Delta_{\mathrm{t,IPC}}$) and rough estimate of the trap density (N_{t}) for the investigated organic solar cells.

Device	$E_{\mathrm{H,D}}$ [eV]	$E_{\mathrm{L,A}}$ [eV]	d [nm]	Δ_{t} [eV]	N_{t} [cm^{-3}]
PBDB-T:ITIC	5.21[15]	3.89[16]	110	0.46	4×10^{16}
PCDTBT:$PC_{70}BM$	5.3[17]	3.9[18]	80	0.48	5×10^{16}
PTB7-Th:$PC_{70}BM$ (w/ DIO)	5.24[19]	3.9[18]	110	0.44	3×10^{16}
PBDB-T:EH-IDTBR	5.21[15]	3.89[16]	110	0.51	4×10^{16}
PTB7-Th:ITIC	5.24[19]	3.89[16]	90	0.38	5×10^{16}
PBDB-T:IT-4F	5.21[15]	4.14[16]	110	0.45	3×10^{16}
PM6:IT-4F	5.45[20]	4.14[16]	110	0.45	3×10^{16}
PM6:Y6	5.45[20]	4.1[21]	100	0.44	4×10^{16}
PM6:BTP-eC9	5.45[20]	4.1[21]	100	0.4	4×10^{16}
PM6:ITIC	5.45[20]	3.89[16]	110	0.46	4×10^{16}
PBDB-T:Y6	5.21[15]	4.1[21]	90	0.42	5×10^{16}
PTB7-Th:$PC_{70}BM$ (w/o DIO)	5.24[19]	3.9[18]	90	0.45	3×10^{16}
PCDTBT:$PC_{70}BM$:m-MTDATA	5.3[17]	3.9[18]	80	0.37	7×10^{17}

Table 2 Parameters used for the drift-diffusion simulations in the main text.

Parameter	Figure 1	PCDTBT:$PC_{70}BM$	PTB7-Th:$PC_{70}BM$	PM6:BTP-eC9
Energy level gap, eV	1.30	1.46	1.31	1.25
Relative permittivity	3.5	3.5	3.5	3.5
Effective density of states, cm^{-3}	10^{20}	10^{20}	10^{20}	10^{20}
Active layer thickness, nm	100	80	110	100
Average generation rate at 1 sun, cm^{-3}s^{-1}	10^{22}	8.0×10^{21}	9.5×10^{21}	1.59×10^{22}
Mobility, cm^{2}V^{-1}s^{-1}	10^{-4}	5×10^{-4}	5×10^{-3}	1.5×10^{-3}
Band-to-band recomb., cm^{3}s^{-1}	10^{-11}	2.6×10^{-10}	5.2×10^{-11}	1.6×10^{-12}
Trap density, cm^{-3}	10^{17}	2.5×10^{16}	8×10^{16}	1.6×10^{17}
Trap depth, eV	0.40	0.49	0.49	0.41
SRH carrier lifetime, µs	1.0	0.31	0.24	4.0
Injection barriers, eV	0.20	0.20	0.0	0.05
Shunt resistance, Ωcm^2	4×10^5	2×10^4	1.7×10^3	7×10^5
Series resistance, Ωcm^2	-	1.0	1.0	1.0

Table 3 Photovoltaic parameters (V_{oc}, FF, J_{sc} and PCE) for the investigated organic solar cells. The statistical data were obtained from over 20 individual devices.

Device	V_{oc} [V]	FF [%]	J_{sc} [mA cm^{-2}]	PCE (PCE_{max}) [%]
PCDTBT:PC_{70}BM	0.90 ± 0.01	63.4 ± 0.5	10.7 ± 0.2	6.2 ± 0.1 (6.4)
PCDTBT:PC_{70}BM:m-MTDATA	0.58 ± 0.01	35.2 ± 1.2	1.5 ± 0.1	0.32 ± 0.1 (0.34)
PTB7-Th:PC_{70}BM w/o DIO	0.70 ± 0.01	44.5 ± 0.7	14.3 ± 0.3	4.5 ± 0.3 (6.1)
PTB7-Th:PC_{70}BM w/ DIO	0.76 ± 0.01	65.3 ± 0.4	17.0 ± 0.3	8.6 ± 0.3 (9.1)
PBDB-T:EH-IDTBR	0.99 ± 0.01	57.7 ± 1.0	10.2 ± 0.2	5.7 ± 0.2 (6.0)
PBDB-T:IT-4F	0.72 ± 0.01	75.0 ± 0.7	18.5 ± 0.4	9.6 ± 0.2 (9.9)
PM6:IT-4F	0.83 ± 0.01	76.0 ± 0.6	19.4 ± 0.4	12.2 ± 0.2 (12.5)
PBDB-T:ITIC	0.90 ± 0.01	68.5 ± 1.1	15.5 ± 0.5	9.3 ± 0.4 (9.7)
PM6:ITIC	0.97 ± 0.01	61.2 ± 1.2	16.2 ± 0.6	9.2 ± 0.3(9.6)
PTB7-TH:ITIC	0.81 ± 0.01	65.3 ± 0.5	14.2 ± 0.3	7.6 ± 0.2(7.9)
PBDB-T:Y6	0.74 ± 0.01	61.4 ± 0.9	24.0 ± 0.4	10.9 ± 0.4 (11.5)
PM6:Y6	0.84 ± 0.01	76.0 ± 1.0	24.2 ± 0.4	15.2 ± 0.3 (15.6)
PM6:BTP-eC9	0.84 ± 0.01	77.5 ± 1.0	24.3 ± 0.4	15.8 ± 0.4 (16.4)

Table 4 Photovoltaic parameters (V_{oc}, FF, J_{sc} and PCE) for a 100 nm thin-film PM6:Y6 solar cell measured under artificial 1 sun light (AM 1.5G condition) with different device areas. The area was changed by varying the pixel size and further corrected with illumination masks.

Pixel Area [mm^2]	V_{oc} [V]	FF [%]	J_{sc} [mA cm^{-2}]	PCE [%]
2.56	0.84 ± 0.01	76.0 ± 0.6	24.3 ± 0.3	15.5 ± 0.2
7.38	0.84 ± 0.01	73.5 ± 0.4	24.1 ± 0.3	14.9 ± 0.2
16.9	0.83 ± 0.01	71..2 ± 0.2	24.2 ± 0.2	14.3 ± 0.2

Supplementary References

1. Selberherr, S. *Analysis and Simulation of Semiconductor Devices*. *Analysis and Simulation of Semiconductor Devices* (Springer Vienna, 1984). doi:10.1007/978-3-7091-8752-4_1

2. W. H. Press, S. A. Teukolsky, W. T. Vetterling & B. P. Flannery. Numerical Recipes in C: The Art of ScientificComputing. *Cambridge Univ. Press. Cambridge* (1992).

3. Burkhard, G. F., Hoke, E. T. & McGehee, M. D. Accounting for interference, scattering, and electrode absorption to make accurate internal quantum efficiency measurements in organic and other thin solar cells. *Adv. Mater.* **22**, 3293–3297 (2010).

4. Pettersson, L. A. A., Roman, L. S. & Inganäs, O. Modeling photocurrent action spectra of photovoltaic devices based on organic thin films. *J. Appl. Phys.* **86**, 487–496 (1999).

5. Sandberg, O. J. *et al.* Impact of a Doping-Induced Space-Charge Region on the Collection of Photogenerated Charge Carriers in Thin-Film Solar Cells Based on Low-Mobility Semiconductors. *Phys. Rev. Appl.* **12**, 034008 (2019).

6. Zarrabi, N. *et al.* Charge-generating mid-gap trap states define the thermodynamic limit of organic photovoltaic devices. *Nat. Commun.* **11**, 5567 (2020).

7. Vandewal, K., Tvingstedt, K., Gadisa, A., Inganäs, O. & Manca, J. V. Relating the open-circuit voltage to interface molecular properties of donor:acceptor bulk heterojunction solar cells. *Phys. Rev. B - Condens. Matter Mater. Phys.* **81**, 1–8 (2010).

8. Yan, Y. *et al.* Correlating Nanoscale Morphology with Device Performance in Conventional and Inverted PffBT4T-2OD:PC 71 BM Polymer Solar Cells. *ACS Appl. Energy Mater.* **1**, 3505–3512 (2018).

9. Li, N. & Brabec, C. J. Air-processed polymer tandem solar cells with power conversion efficiency exceeding 10%. *Energy Environ. Sci.* **8**, 2902–2909 (2015).

10. Kim, W. *et al.* Conflicted effects of a solvent additive on PTB7:PC71BM bulk heterojunction solar cells. *J. Phys. Chem. C* **119**, 5954–5961 (2015).

11. Pearson, A. J. *et al.* Critical light instability in CB/DIO processed PBDTTT-EFT:PC71BM organic photovoltaic devices. *Org. Electron.* **30**, 225–236 (2016).

12. Foertig, A. *et al.* Nongeminate and geminate recombination in PTB7:PCBM Solar Cells. *Adv. Funct. Mater.* **24**, 1306–1311 (2014).

13. Zuo, G. *et al.* Molecular Doping and Trap Filling in Organic Semiconductor Host-Guest Systems. *J. Phys. Chem. C* **121**, 7767–7775 (2017).

14. Zuo, G., Linares, M., Upreti, T. & Kemerink, M. General rule for the energy of water-induced traps in organic semiconductors. *Nat. Mater.* **18**, 588–593 (2019).

15. Zhao, W., Li, S., Zhang, S., Liu, X. & Hou, J. Ternary Polymer Solar Cells based on Two Acceptors and One Donor for Achieving 12.2% Efficiency. *Adv. Mater.* **29**, (2017).

16. Zhao, W. *et al.* Molecular Optimization Enables over 13% Efficiency in Organic Solar

Cells. *J. Am. Chem. Soc.* **139**, 7148–7151 (2017).

17. Ratcliff, E. L. *et al.* Energy level alignment in PCDTBT:PC70BM solar cells: Solution processed NiOx for improved hole collection and efficiency. *Org. Electron.* **13**, 744–749 (2012).

18. Tan, Z. *et al.* High performance polymer solar cells with as-prepared zirconium acetylacetonate film as cathode buffer layer. *Sci. Rep.* **4**, 1–9 (2015).

19. Zhang, S. *et al.* Side chain selection for designing highly efficient photovoltaic polymers with 2D-conjugated structure. *Macromolecules* **47**, 4653–4659 (2014).

20. Zhang, H. *et al.* Over 14% Efficiency in Organic Solar Cells Enabled by Chlorinated Nonfullerene Small-Molecule Acceptors. *Adv. Mater.* **30**, 1–7 (2018).

21. Yuan, J. *et al.* Single-Junction Organic Solar Cell with over 15% Efficiency Using Fused-Ring Acceptor with Electron-Deficient Core. *Joule* **3**, 1140–1151 (2019).
