# Supplementary material for: Direct observation of trap-assisted recombination in organic photovoltaic devices
Source: Nat Commun. 2021 Jun 14;12:3603. doi: 10.1038/s41467-021-23870-x (PMC8203604; doi:10.1038/s41467-021-23870-x)
Supplement: Supplementary file 3 — Solar Cells Reporting Summary [file 41467_2021_23870_MOESM3_ESM.pdf]

nature research

Corresponding author(s): Ardalan Armin, Oskar J. Sandberg

Solar Cells Reporting Summary

Nature Research wishes to improve the reproducibility of the work that we publish. This form is intended for publication with all accepted papers reporting the characterization of photovoltaic devices and provides structure for consistency and transparency in reporting. Some list items might not apply to an individual manuscript, but all fields must be completed for clarity.

For further information on Nature Research policies, including our data availability policy, see Authors & Referees.

Experimental design

Please check: are the following details reported in the manuscript?

1. Dimensions

Item	Yes/No	Response
Area of the tested solar cells	[x] Yes [ ] No	2.56, 7.38 and 16.9 mm^2. See Methods and device fabrication section in Supplementary Information.
Method used to determine the device area	[x] Yes [ ] No	See Methods. Combination of illumination mask and pixel size.

2. Current-voltage characterization

Item	Yes/No	Response
Current density-voltage (J-V) plots in both forward and backward direction	[ ] Yes [x] No	See the captions of Fig S3 and Fig S9. No hysteresis observed.
Voltage scan conditions<br>*For instance: scan direction, speed, dwell times*	[x] Yes [ ] No	Information provided in the Methods section in the main text. 0.5V/s.
Test environment<br>*For instance: characterization temperature, in air or in glove box*	[x] Yes [ ] No	See Methods. Encapsulated devices were tested in air and room temperature.
Protocol for preconditioning of the device before its characterization	[x] Yes [ ] No	See Methods. OPV devices were tested right after fabrication.
Stability of the J-V characteristic<br>*Verified with time evolution of the maximum power point or with the photocurrent at maximum power point; see ref. 7 for details.*	[ ] Yes [x] No	OPV devices exhibit no short term instability in the JV characteristics.

3. Hysteresis or any other unusual behaviour

Item	Yes/No	Response
Description of the unusual behaviour observed during the characterization	[ ] Yes [x] No	No unusual behavior or hysteresis observed. See the captions of Fig S3 and Fig S9.
Related experimental data	[ ] Yes [x] No	No unusual behaviour observed.

4. Efficiency

Item	Yes/No	Response
External quantum efficiency (EQE) or incident photons to current efficiency (IPCE)	[x] Yes [ ] No	See Supplementary Information (Figure S10).
A comparison between the integrated response under the standard reference spectrum and the response measure under the simulator	[x] Yes [ ] No	See Supplementary Information (Figure S10).
For tandem solar cells, the bias illumination and bias voltage used for each subcell	[ ] Yes [x] No	N/A

5. Calibration

Item	Yes/No	Response
Light source and reference cell or sensor used for the characterization	[x] Yes [ ] No	See Methods. Calibrated silicon solar cells, Newport.
Confirmation that the reference cell was calibrated and certified	[x] Yes [ ] No	Newport.

Calculation of spectral mismatch between the reference cell and the devices under test

☐ Yes
☒ No

See the caption of Fig S10. Integrated EQE and Jsc are within 5% agreement.

6. Mask/aperture

Size of the mask/aperture used during testing

☒ Yes
☐ No

See Methods

Variation of the measured short-circuit current density with the mask/aperture area

☒ Yes
☐ No

See Supplementary Information (Table S4).

7. Performance certification

Identity of the independent certification laboratory that confirmed the photovoltaic performance

☐ Yes
☒ No

This work does not report any record efficiency and the solar cells are not certified. The efficiencies are in agreement with the exisiting litrature.

A copy of any certificate(s)
Provide in Supplementary Information

☐ Yes
☒ No

N/A

8. Statistics

Number of solar cells tested

☒ Yes
☐ No

See Supplementary Information Table S3. 20 Pixels per device.

Statistical analysis of the device performance

☒ Yes
☐ No

See Supplementary Information (Table S3).

9. Long-term stability analysis

Type of analysis, bias conditions and environmental conditions
For instance: illumination type, temperature, atmosphere humidity, encapsulation method, preconditioning temperature

☐ Yes
☒ No

Long-term stability analysis was not subject of our work.
